# Calcinosis cutis of the lower legs – hyperphosphatemic familial tumoral calcinosis in a patient with GALNT3 mutation

**DOI:** 10.1111/ddg.15716

**Published:** 2025-05-02

**Authors:** David Ranzinger, Tobias Schaefer, Franziska Schauer, Kilian Eyerich, Anna Caroline Pilz

**Affiliations:** ^1^ Department of Dermatology Medical Center University of Freiburg Freiburg Germany; ^2^ Renal Division Medical Center University of Freiburg Freiburg Germany

Dear Editors,

We report on a 39‐year‐old European male patient, who presented to our outpatient clinic with subcutaneous indurations of both lower legs. These lesions were primarily located on the ventral and medial of the lower legs, had first been noticed by the patient approximately three years earlier, and caused pulling pain when pressure was applied (Figure 1). His medical history included left‐sided renal agenesis and arterial hypertension, with no other reported conditions or family history of skin diseases.

**FIGURE 1:**
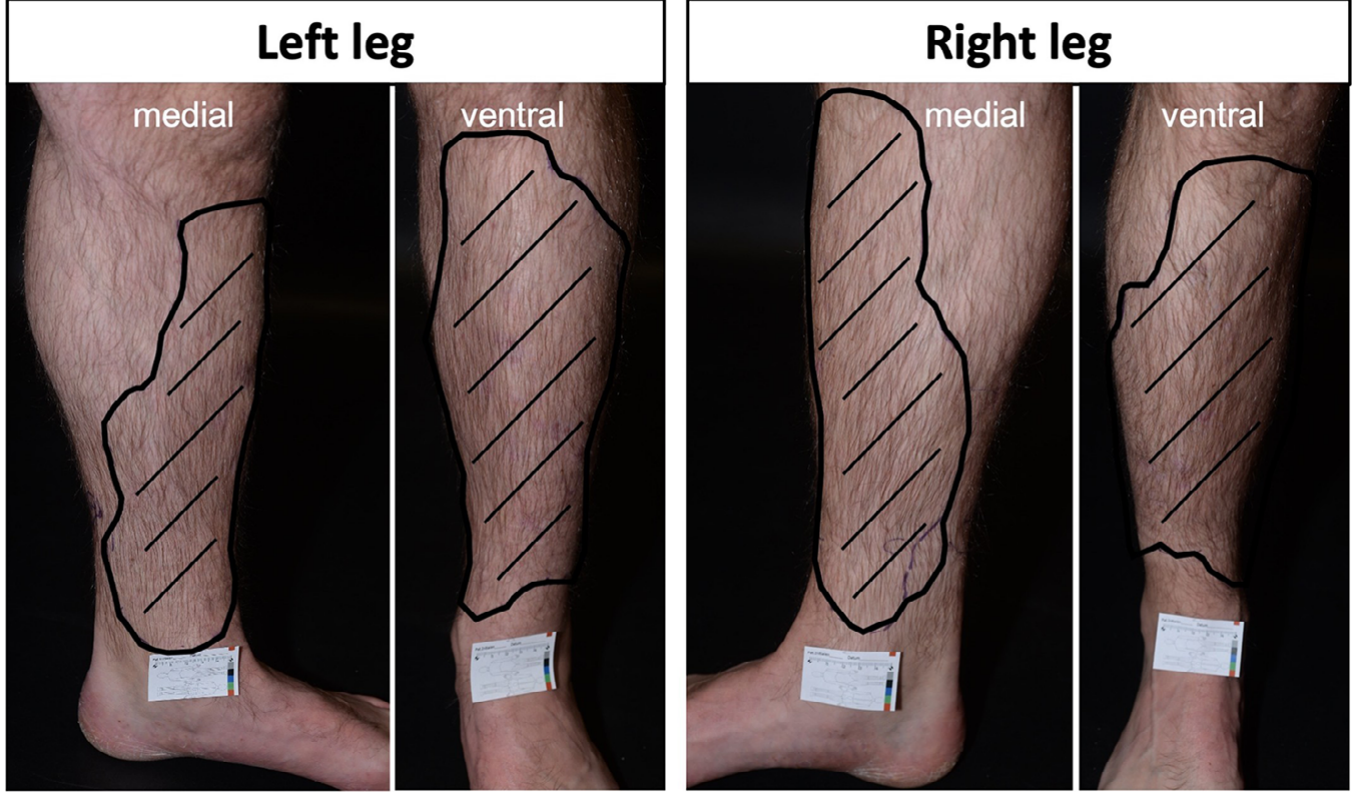
Extent of the subcutaneous calcifications of the left and right lower leg indicated by the circled dashed area.

Histological examination revealed amorphous calcium precipitates at the transition from dermis to subcutis and within the subcutis. There were no signs of inflammation.

The causes of calcinosis cutis, and thus its differential diagnoses, are manifold. Dystrophic variants are triggered, for example, by hereditary and/or autoimmune connective tissue diseases such as systemic sclerosis or dermatomyositis and metastatic calcinosis cutis results from abnormal calcium and/or phosphate metabolism.

Laboratory tests revealed elevated phosphate levels at 1.8 mmol/l (reference range: 0.81–1.45 mmol/l) and normal calcium levels at 2.49 mmol/l (reference range: 2.09–2.56 mmol/l). Parathyroid hormone levels were in the lower normal range at 22 pg/ml (reference range: 15–65 pg/ml). Renal function was normal and no significant proteinuria was detected. Assessment of the tubular function indicated a tubular reabsorption of phosphate close to 100% (80%–95%). Consequently, hyperphosphatemic familial tumoral calcinosis (HFTC) was suspected. To confirm this, whole exome sequencing was performed from peripheral blood and a homozygous mutation in the NM_004482.4 (*GALNT3*) gene was identified.

Hyperphosphatemic familial tumoral calcinosis is an exceptionally rare disease, it predominantly affects individuals of Middle Eastern or African‐American descent,^1^ and is caused by resistance to or a deficiency of fibroblast growth factor 23 (FGF23), a hormone that regulates phosphate levels in the body.^2^

Disorders involving FGF23 lead to hyperphosphatemia due to increased renal tubular phosphate reabsorption, while serum calcium levels and renal function remain unaffected.^3^ Underlying mutations are found in the *FGF23* gene, the *KL* gene encoding Klotho, an essential co‐receptor for FGF23 signaling, and most commonly in the *GALNT3* gene. *GALNT3* encodes N‐acetylgalactosaminyltransferase 3, an enzyme that catalyzes the O‐glycosylation of proteins and plays a crucial role in regulating FGF23 activity.^4^

In our patient's genome a homozygous c.516‐2A>G mutation in the *GALNT3* gene was identified. The transition from adenine to guanine is located in the acceptor splice site of intron 1. So far, this mutation has only been reported once in literature by Masi et al.^5^ With the help of a computational prediction tool, Masi et al. forecasted that the mutation leads to a skipping of exon 2 during the splicing process (Figure 2).^5^

**FIGURE 2:**
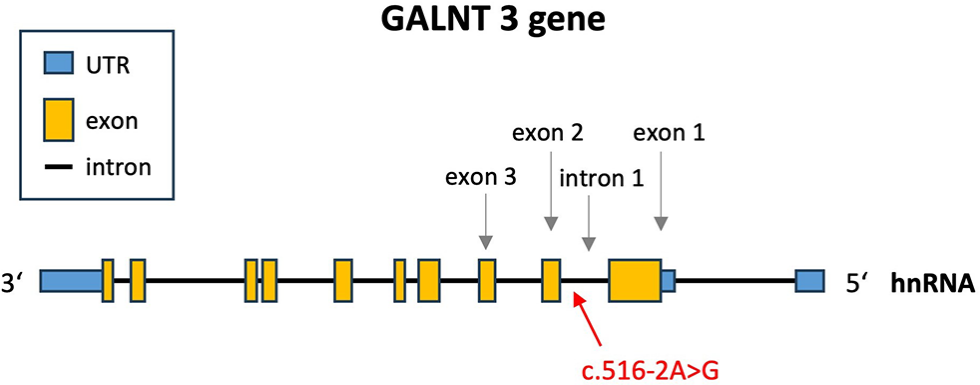
Transition of adenine to guanine in the acceptor splice site of intron 1.

Other authors described an adenine to thymine exchange in the same location with Laleye et al. demonstrating experimentally that the A>T mutation indeed results in a skipping of exon 2 and a frame shift.^2^, ^6^, ^7^ This causes a premature stop‐codon after four amino acids in exon 3, leading to a truncated, likely non‐functional, protein.^7^ A similar outcome may be expected for the A>G transition.

The clinical presentation of hyperphosphatemic familial tumoral calcinosis is highly variable, but tumoral calcifications typically occur in areas of repetitive trauma or pressure (e.g., elbows, shoulders, hips, and knees) within the first two decades of life.^2^ Calcifications can also involve extracutaneous tissues, including the testes, dura, kidneys, eyelids, retina (angioid streaks), and blood vessels (vascular calcifications).^8^, ^9^ Additionally, hyperostosis of the diaphyses and dental alterations (e.g., root shortening, pulp obliteration) have been reported.^2^

In our patient, a multiregional computed tomography scan revealed soft tissue calcifications in the subgaleal region, dorsal to the olecranons, and on the dorsum of the feet, along with arteriosclerotic changes in the aorta, pelvic vessels, forearms, and lower legs (Figure 3). Corneal calcium deposits were identified, whereas no dental abnormalities were detected.

**FIGURE 3:**
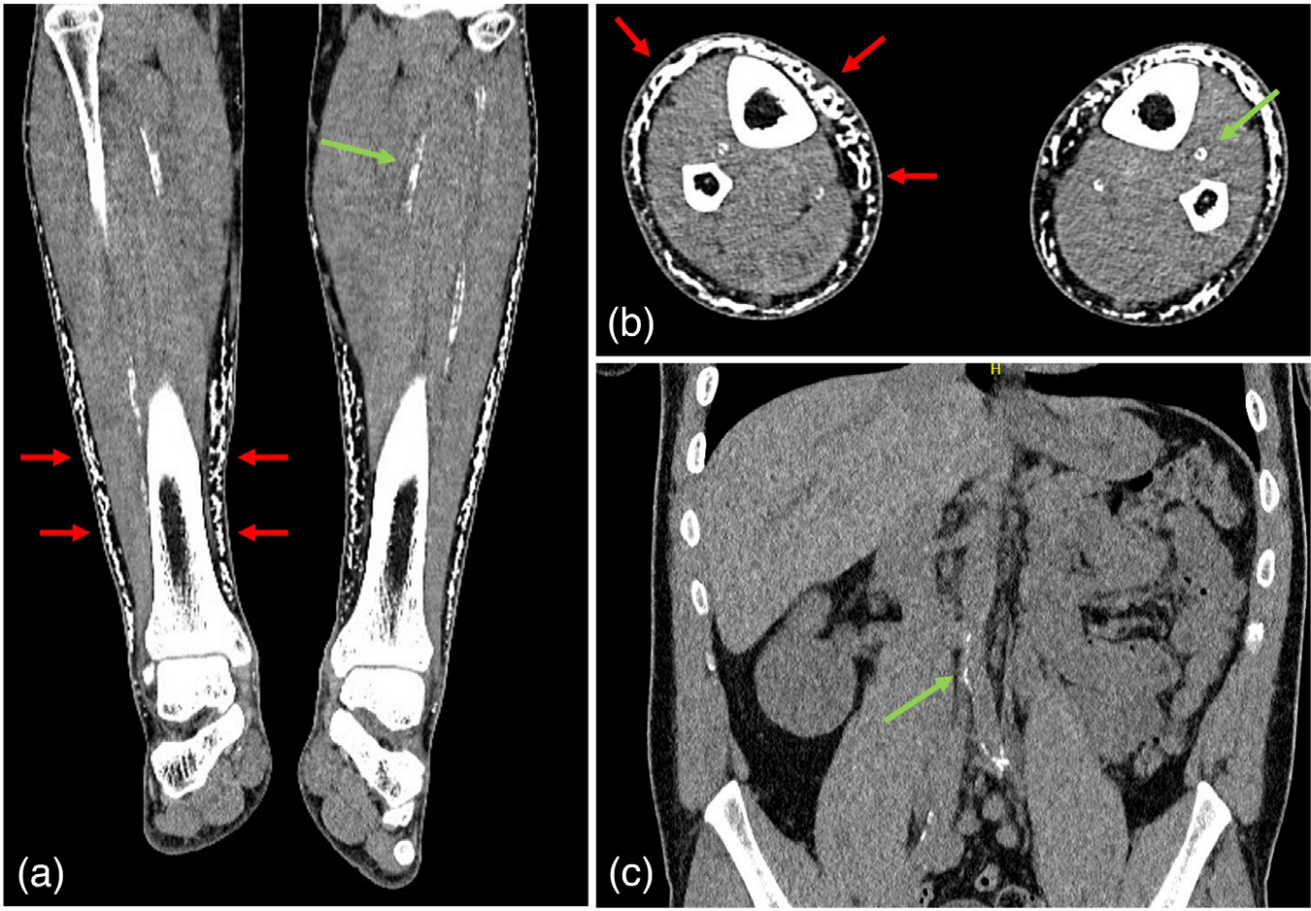
Lower legs (a) in frontal and (b) transverse section. (c) Abdomen in frontal section. Subcutaneous calcifications of the lower legs affecting partly the total circumference (red arrows). The arterial vessels of the lower legs and the abdominal aorta with the descending iliac vessels show pronounced calcifications (green arrows).

Our patient was initially treated with a low‐phosphate diet and 2.4 g of the phosphate binder sevelamer carbonate daily. As phosphate levels did not drop after 6 months, the dose was increased to 4.8 g per day. Arterial hypertension was treated with ramipril, and no intervention was needed for the ophthalmologic involvement. Since follow‐up appointments in our department were missed, no assessment of the development of the calcifications was possible.

This report presents the second case of HFTC with a c.516‐2A>G mutation in the *GALNT3* gene. In contrast to the majority of reported cases of hyperphosphatemic familial tumoral calcinosis, both our patient and the case described by Masi et al. are of Caucasian origin.

Both were mainly affected at the lower extremities and showed intracranial and vascular calcifications, but neither had dental alterations and/or angioid streaks. Therefore, the c.516‐2A>G mutation might be predominately found in Caucasians and might lead to a particular HFTC phenotype.

## CONFLICT OF INTEREST STATEMENT

None.
